# The Book World of Medicine and Science

**Published:** 1894-05-26

**Authors:** 


### The Book World of Medicine and Science.

### BOOK REVIEWS.

The Cyclist Year-book, 1894. Edited by Henry Sturmey. (Iliffe and Son, 3, St. Bride Street, Ludgate Circus, London.)

It may be reasonably doubted whether any form of active recreation which the world has ever seen has claimed so many and such enthusiastic votaries as cycling. Every month one hears of new recruits, new clubs, new records; and as constantly the trade continues to pour its hordes of new machines, good and bad, upon that ever-growing section of humanity which prefers progression on wheels to that on legs. It is for the benefit of those who are interested in cycling in its varied aspects that the year-book is brought out, and it is full of miscellaneous information, from cover to cover, on all things that concern the wheelman's world. Here are chronicled the "records" of various kinds which were made during the last year, as well as details of the machines, tyres, &c., by which the work of the twelve months has been effected. Not the least amusing page of the volume is that containing a list of the dead clubs, and of those who, if they are not dead, do not owe their vitality to the promptitude of their secretaries. These are, however, only a few points selected at random from a work which has been compiled with considerable care, and which must prove of much interest as well as use to every lover of road or track who cares to be kept *au courant* with the doings of the cyclist world.

The Medical Annual, 1894. (Bristol: John Wright and Co. Pp. 686. Price 7s. 6d.)

This book is a great advance on anything that has gone before, both in its contents and its illustrations. It is divided into three parts—therapeutics, new treatment, and miscellaneous, comprising sanitary science, new inventions, and directories to institutions, asylums, &c. Under therapeutics a good sketch is given of the various new remedies which have been introduced during the year, but the great mass of the book is devoted to new treatment. Here we have something much more than a mere catalogue of new methods. In some departments the year seems to have been comparatively barren, and where this is the case a line or two, with a reference to what has been done, is found sufficient; but where special advance has been made the editors have not contented themselves with inserting only the novel points, but have obtained from their contributors valuable essays on the whole subject, as, for example, in the case of diseases of the ear, to which thirty pages are devoted, together with many illustrations. Another thirty pages, and a large number of admirable illustrations, are given up to an article on "Facial Expression in Insanity." Sir Alfred Garrod contributes a short but useful article on the "Differential Diagnosis of Rheumatoid Anthritis and Gout," to which he also adds a *resumé* of the non-articular manifestations of the latter disease. It is to be noted that in some instances where somewhat opposite views may be taken in regard to a method of treatment, more than one article is inserted on the same subject. The surgery of the spinal cord, for example, is at present *sub judice*, and somewhat diverse views are taken in different quarters as to the propriety of trephining or laminectomy. We find, therefore, that on this subject there are separate articles—one by Professor Sonnenburg, of Berlin; the other by Mr. William Thorburn, assistant surgeon to the Royal Infirmary, Manchester. In the article on "Diseases of the Eye," by Mr. Simeon Snell, there is an excellent and well-illustrated chapter on cataract extraction, giving with much minuteness the different steps of the operation as at present performed. There is also a *resumé* of the methods of antisepsis employed by various surgeons. There can be no doubt that, as a presentment of the year's advance, this "Medical Annual" takes a high place, and that, even as a mere index to the original communications on the various subjects, it forms a most useful addition to the practitioner's library.

Cimiez: Its Health and Climate. By H. Evelyn Crook, M.D. (London: McCorquodale and Co. 1894.)

In thirty pages Dr. Crook gives a sketch of the general climate of the Western Riviera, and more especially of that of Cimiez. This town, standing on the site of the ancient city of Cemenelion, is now in reality a suburb of Nice, from which it is distant only about two miles. It has the advantage of standing on sandy gravel, raised two to three hundred feet above the level of the sea, on a hill which juts out like a promontory into the plain in which Nice stands. It has, then, the same protection from the north winds, by the mountains behind it, as is enjoyed by Nice; and while its elevated position renders it free from sea fogs, the mountain mists are said never to descend to so low a level. Much stress is laid on the sandy, porous nature of the soil, which enables the roads and paths to dry up very quickly, even after the heavy rains which are one of the climatic features of the Western Riviera. As in most medical books on special health resorts, one feels that there is a desire to make the most of everything which can be said in favour of the particular place in question. Nevertheless, we have not the slightest doubt that Cimiez, with its elevated position, is a charming place, especially as we are assured that the water supply is brought from a considerable distance, and is of undoubted purity. The house drainage, we are informed, is carried to cesspools dug at suitable distances from each dwelling, and no doubt the isolation so provided is not without its advantages. On the other hand, the fact that the road drains for the surface and storm waters enter the sewers of Nice is not very reassuring, an elevated district being very apt in dry weather, when gully traps are empty, to act as a foul air outlet to the town sewers. We should have liked to hear more about the drives and walks and other facilities for lightening the tedium of illness; but even as it stands, the book will doubtless serve its purpose in drawing attention to a health resort which is well worthy of consideration by those who intend to winter in the sunny south.

Electricity in Diseases of Women and Obstetrics. By Dr. Franklin Martin. (Chicago: The Keener Company.)

Electricity in diseases of women is still viewed with more than suspicion by the majority of medical men. It is such an easy refuge for the obstetric physician "who does not operate." It is expensive, too, for the patient; for the number of *séances* must be many if electricity be used at all. The present work is an attempt to raise the cloud of mystery with which it is surrounded, and to explain its action scientifically. It aims at showing how accuracy in administration of the dose may be attained, and it endeavours to explain how electricity causes the absorption of fibroid growths. The author fails, however, to convince us of the selective action of electricity for morbid tissues. We have seen abdomens opened for the removal of fibroid tumours by hysterectomy, both when electricity has been previously used and when it has not. In the former we have seen adhesions so numerous that the dissection of the uterus from the surrounding parts occupied nearly three hours. We have never seen anything like this when electricity has not been used. Such cases as these make us doubt very much whether currents sufficiently strong toaffect the tumour can possibly be harmless to the surrounding healthy tissues through which they must pass. We doubt again, too, the possibility in the case of a fibroid of any size, of cauterising the whole interior of the uterus, including the surface of the growth. We have given this book our very careful consideration, but we must confess to remaining sceptical as to the advantages of electricity in the treatment of uterine diseases.

